# Items

**Published:** 1900-07

**Authors:** 


### ITEMS.

Of Interest to the Beginner.—The office equipment of the late Dr. George S. Palmer is offered for sale, as will be noted by reference to advertising page xxx. A physician beginning practice who needs books, electric batteries, and office furniture will find at 325 Franklin Street much that he stands in need of at low prices.

---

The New York Pharmacal Association has prepared for distribution to the medical profession a handsome and artistic *perpetual* calendar, which is now ready for mailing. Instead of presenting a calendar at the beginning of the year, according to the usual custom, this company prefers the season when the physician is not deluged with all sorts and conditions of chronological recorders, and is thus better enabled to welcome and appreciate such an addition to his office. The new lactopeptine perpetual calendar is not intended for hanging upon the wall, but to stand upon the doctor's desk, and for this reason has a strong easel back to support it. The coloring is exceedingly soft and attractive, consisting of delicate shades of lavender, purple, crushed strawberry, and buff-yellow. The few words relative to lactopeptine are entirely unobtrusive, and do not interfere in the least with the general artistic effect. In the near foreground on either side are two gracefully-draped female figures with flowing hair; around the edges appear the various signs of the zodiac.

One of these calendars will be sent to any physician who may request same.

